# The complete mitochondrial genome of the Brambling *Fringilla montifringilla* (Passeriformes: Fringillidae) from Maor Mountain, China

**DOI:** 10.1080/23802359.2022.2158693

**Published:** 2023-01-02

**Authors:** Shuang Cui, Wei Yu, Dehuai Meng, Junda Chen, Xu Zhang, Xuyang Zhao, Liwei Teng, Zhensheng Liu

**Affiliations:** a College of Wildlife And Protected Area, Northeast Forestry University, Harbin, China; b Key Laboratory of Conservation Biology, National Forestry And Grassland Administration, Harbin, China

**Keywords:** Complete mitochondrial genome, *Fringilla montifringilla*, Brambling

## Abstract

The Brambling (*Fringilla montifringilla*) (Linnaeus 1758) is a member of the Passeriformes family of birds and primarily feeds on grass seeds and grains. Muscle tissue was collected from birds sampled from Moar Mountain, China, and the complete mitochondrial genome was sequenced. Its mitochondrial genome consists of 13 protein-coding genes (PCGs), 2 rRNA genes (12S rRNA and 16S rRNA), 22 tRNA genes, and 1 control region (CR). The genome comprises 30.30% A, 23.32% T, 14.31% G, and 32.07% C bases. Phylogenetically, *F. montifringilla* is closely related to the *Fringilla coelebs*, *Fringilla teydea teydea* and *Fringilla polatzeki*.

## Introduction

The Brambling (*Fringilla montifringilla*) (Linnaeus 1758), a member of the Passeriformes family of birds, a very large and diverse family commonly known as perching birds, and is also known as the Tiger-skin Brambling (Liu et al. 2004; Fang et al. 2008). Large population numbers occupy a very wide geographical range and inhabit large areas of the northern Asian continent (MacKinnon and Phillipps 2002), primarily feeding on grass seeds and grains (Liu et al. 2004). Although the population size is declining, the rate of decline is slow, and it is classified as a species of Least Concern on the Red Data List (Vikan et al. 2010).

Currently, *F. montifringilla* research focuses on energetics and thermoregulation (Liu et al. 2004), vocalization (Jiang et al. 2001; Zhao et al. 2003), sex ratio and age composition (Liu and Liu 2021; Jenni 2022; Kumar et al., 2016), migration (Fang et al. 2008), and evolution of defenses (Vikan et al. 2010). Evolutionary analysis and data on the species’ mitochondrial genome are limited. To date, more than 569 mitochondrial genomes have been sequenced and stored on Genbank as part of the BioProject initiative. In this study, we sequenced the complete mitochondria of *F. montifringilla* and analyzed its phylogenic relationship to other birds of the same genus. This data is of reference significance for the protection of and subsequent research on the Brambling.

## Materials and methods

**Figure 1.**
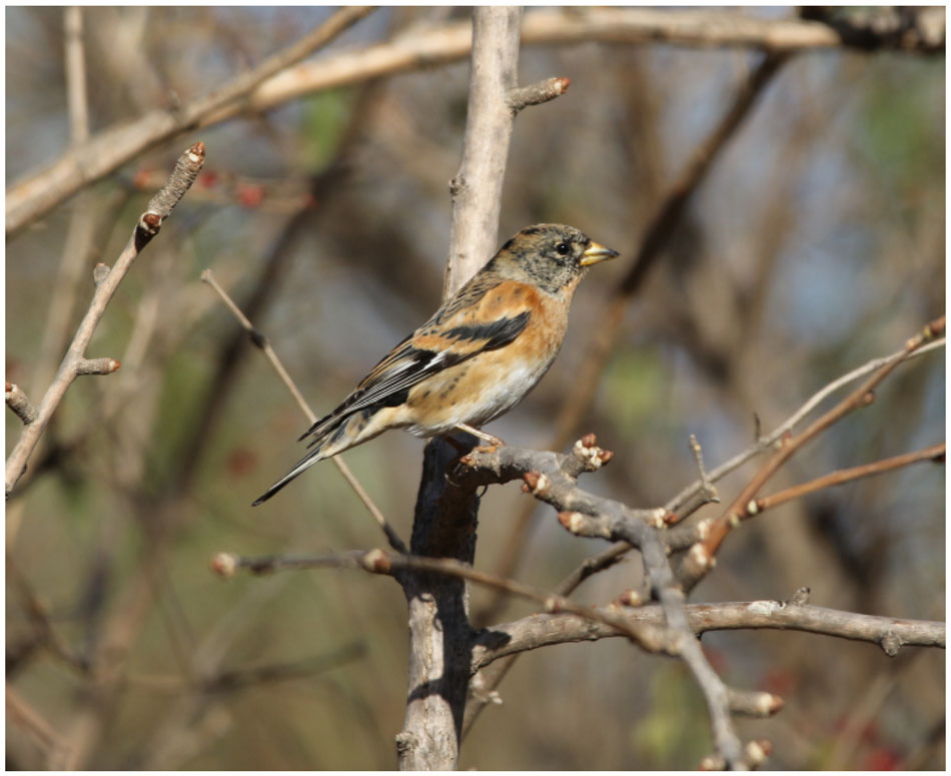
The *F. montifringilla* reference image. This image was taken by Dr. Meng Dehuai using a Canon 700 D camera in Maor Mountain, China (127°30′–127°34′E, 45°20′–45°25′N).

The mitochondrial genome of *F. montifringilla* was sequenced from muscle tissue collected from Maor Mountain in China (127°30′–127°34′E, 45°20′–45°25′N). After use, specimens were deposited at Northeast Forestry University’s College of Wildlife and Protected Areas, voucher number YQ202106 (Zhensheng Liu, Email: zhenshengliu @163.com) (Figure 1).

Illumina HiSeq sequencing technology was used to perform paired-end (PE) sequencing using the whole-genome shotgun (WGS) method and next-generation sequencing (NGS). DNA was extracted from samples, followed by purification, database building, and sequencing, and the resulting readable Raw Data was uploaded to GenBank as SRR17012662. The complete genome was submitted to GenBank under accession number OL629048.1.

A maximum likelihood analysis was performed in MEGA7 (Kumar et al. 2016) to infer phylogenetic relationships (Rogers and Swofford 1998; Chen et al. 2022).

## Results

**Figure 2.**
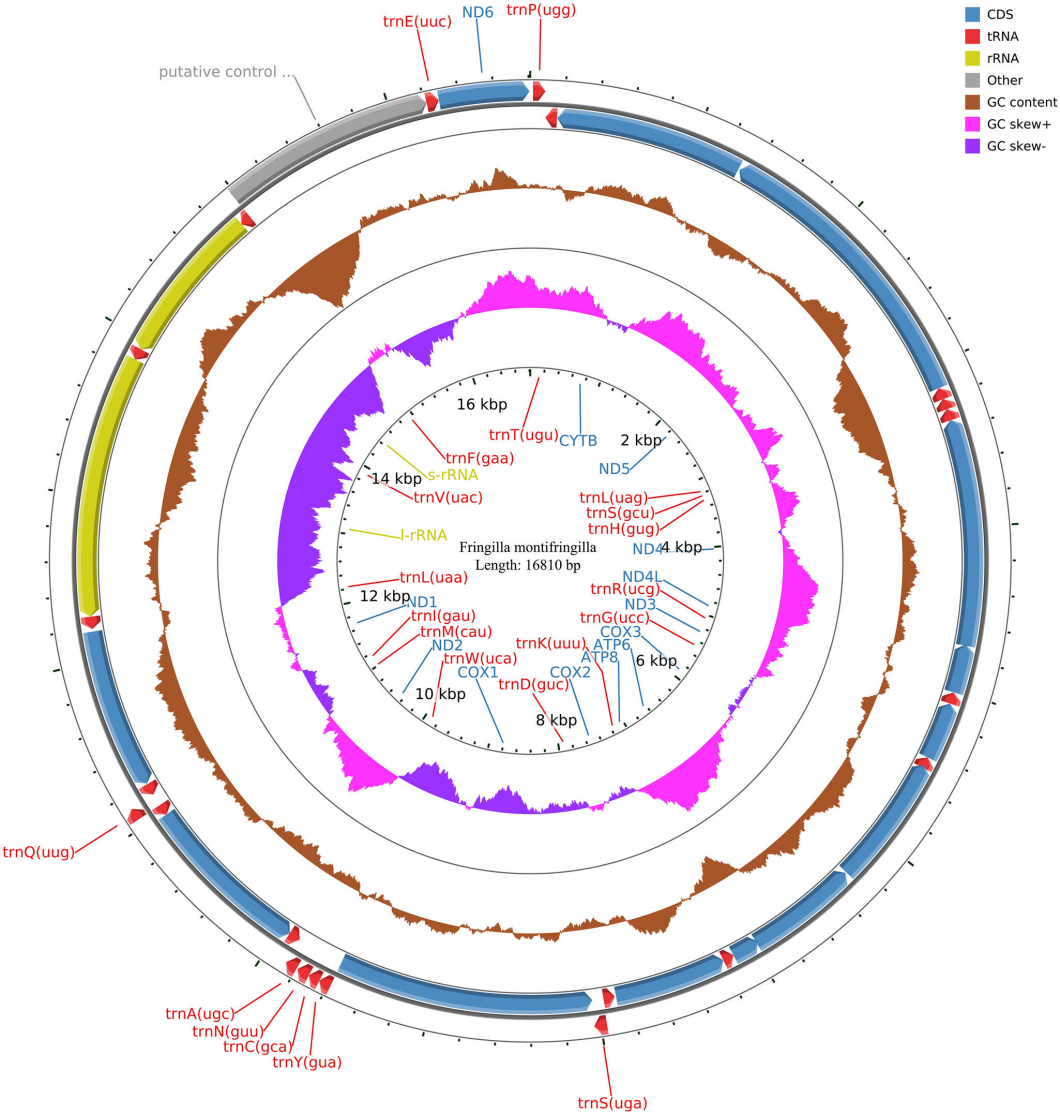
Circular maps of the mitochondrial genome of *F. montifringilla.*

The mitochondrial genome of *F. montifringilla* consists of 13 protein-coding genes (PCGs), 2 rRNA genes (12S rRNA and 16S rRNA), 22 tRNA genes, and 1 control region (CR). The genome is 16,810 bp long, with a GC content of 46.38%. The genome consists of 30.30% A, 23.32% T, 14.31% G, and 32.07% C bases. The total length of the 13 protein-coding genes is 11,413 bp. Besides the initiation codon, 11 are ATG (ND1, ND2, ND4, ND4L, ND5, ND6, COX2, COX3, ATP6, ATP8, and CYTB), one is ATT (ND3), and one is GTG (COX1). There were nine stop codons with TAA (ND2, ND3, ND4, ND4L, COX2, COX3, ATP6, ATP8, and CYTB), two with AGG (COX1, ND1), one with AGA, and one with TAG (ND6, ND5). All the tRNAs comprised 1562 base pairs. The two rRNA genes comprised 1598 base pairs (12 s rRNA) and 976 base pairs (16 s rRNA), with a control region of 1233 base pairs (Figure 2).

**Figure 3.**
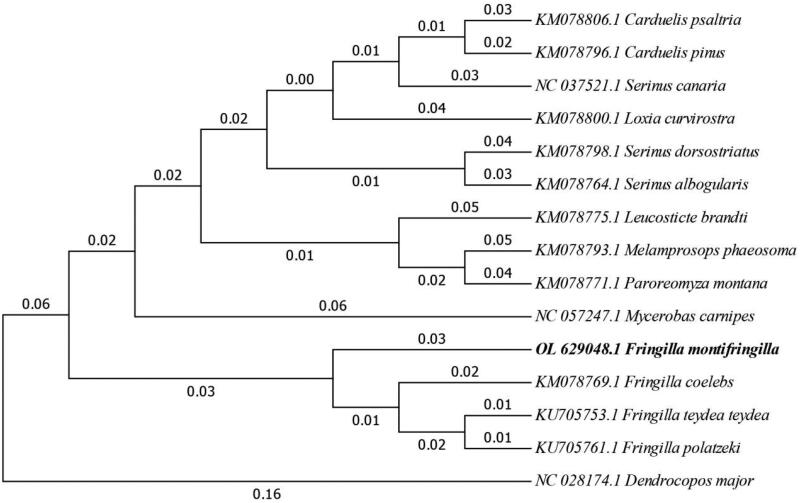
Maximum likelihood consensus tree (1000 bootstrap replicates) constructed from complete mitochondrial genome analyses of *F. montifringilla* and 14 other related species.

The bootstrap consensus tree inferred from 1000 replicates is used to represent the evolutionary history of the taxa analyzed (Felsenstein 1985). As shown in the bootstrap consensus tree (Figure 3), *F. montifringilla* is closely related to the *Fringilla coelebs*, *Fringilla teydea teydea* and *Fringilla polatzeki*.

## Discussion

The population size of *F. montifringilla* is decreasing, although slowly (Lindstrom 1987). This is possibly due to the breeding site tenacity of this species, where ringing recovery studies indicate that the Bramblings breed at sites that are up to 600 km apart in different years (Mikkonen 1983). Despite this tenacity, it is important to sequence the complete mitochondrial genome of this species for reference significance and the protection of subsequent research on the Brambling.

In recent years, research on the phylogeny and evolution of passerines has attracted much attention. For example, Bayesian analysis was used to study the taxonomic status of this subfamily, Subfamily name, of birds revealing a heterogeneous origin of the *Fringilla genus* (Antonio et al. 2007). Additionally, mitochondrial data is often used to infer phylogenetic relationships in these birds. Guo et al. (2007) analyzed the mitochondrial cytochrome b gene sequence of 18 passerine birds to infer their phylogeny. The mitochondrial genome sequence of *F. montifringilla* obtained in this study will provide useful genetic data for further phylogenetic and evolutionary analysis.

## Data Availability

The data supporting this study’s findings are freely available on the NCBI website at https://www.ncbi.nlm.nih.gov/, reference number OL629048.1. The associated BioProject, SRA, and Bio-Sample numbers are PRJNA782766, SRR17012662, and SAMN23394671, respectively.
